# Implementation of the International Association of Diabetes and Pregnancy Study Groups Criteria: Not Always a Cause for Concern

**DOI:** 10.1155/2015/754085

**Published:** 2015-12-28

**Authors:** Pooja Sibartie, Julie Quinlivan

**Affiliations:** ^1^Department of Obstetrics and Gynaecology, Joondalup Health Campus, Joondalup, WA 6027, Australia; ^2^Institute for Health Research, University of Notre Dame Australia, Fremantle, WA 6160, Australia

## Abstract

*Background*. Controversy surrounds the decision to adopt the International Association of Diabetes and Pregnancy Study Groups (IADPSG) criteria for the diagnosis of gestational diabetes mellitus (GDM) as fears that disease prevalence rates will soar have been raised.* Aims*. To investigate the prevalence of pregnancy complicated with GDM before and after the introduction of the IADPSG 2010 diagnostic criteria.* Materials and Methods*. A prospective audit of all women who delivered from July 1, 2010, to June 30, 2014, in a predefined geographic region within the North Metropolitan Health Service of Western Australia. Women were diagnosed with GDM according to Australian Diabetes in Pregnancy Society (ADIPS 1991) criteria until December 31, 2011, and by the IADPSG 2010 criteria after this date. Incidence of GDM and predefined pregnancy outcomes were audited.* Results*. Of 10,296 women, antenatal oral glucose tolerance test (OGTT) results and follow-up data were obtained for 10,103 women (98%), of whom 349 (3.5%) were diagnosed with GDM. The rate of GDM utilising ADIPS criteria was 3.4% and the rate of utilising IADPSG criteria was 3.5% (*p* = 0.92).* Conclusion*. IADPSG diagnostic criteria did not significantly increase the incidence of GDM in this low prevalence region.

## 1. Introduction

Gestational diabetes mellitus (GDM) is a common medical complication of pregnancy defined as “any degree of glucose intolerance with onset or first recognition during pregnancy” [1, 2]. The initial criteria for diagnosis were established more than 40 years ago [3]; however, these criteria did not necessarily identify pregnancies with increased risk of adverse pregnancy outcome [4].

The hyperglycaemia and adverse pregnancy outcome (HAPO) study was conducted to clarify the associations between maternal hyperglycaemia and adverse outcomes. The study showed associations between increasing levels of fasting blood glucose (FBG), 1-hour and 2-hour plasma glucose obtained following an oral glucose tolerance test (OGTT), and birthweight >90th centile and cord-blood serum C-peptide level >90th centile [5]. The secondary outcomes of premature delivery, shoulder dystocia or birth injury, admission to intensive neonatal care unit, hyperbilirubinemia, and preeclampsia were also increased by maternal hyperglycaemia [5].

The consideration of HAPO data led to a recommendation in 2010 by the International Association of Diabetes and Pregnancy Study Groups (IADPSG) for the FBG and 1 h and 2 h glucose levels to diagnose GDM [4]. The diagnostic threshold values were the average glucose values at which the odds for birthweight >90th centile, cord C-peptide >90th centile, and percent body fat >90th centile reached 1.75 times the estimated odds of the outcomes at mean glucose values [4, 5].

However, concern has been expressed that adoption of the new diagnostic criteria would lead to a dramatic increase in the incidence of GDM. One Australian study reported that the change in diagnostic criteria from the previously utilised Australasian Diabetes in Pregnancy Society (ADIPS) 1999 criteria [6] to the new IADPSG 2010 criteria [4] would increase the prevalence of GDM from 9.6% to 13.0% [7]. A NZ study reported that the incidence might rise from 6% to 10% [8]. National debate continues on the workforce implications of the revised criteria and their clinical impact [9, 10].

The aim of this study was to audit the impact of the change from the ADIPS 1999 criteria [6] to the IADPSG 2010 diagnostic criteria [4] within a geographically defined region.

## 2. Methods

A prospective audit of all pregnancies diagnosed with GDM commenced from July 1, 2010, following publication of HAPO and the IADPSG recommendations. The Institutional Ethics Committee determined that the project fulfilled the criteria of an audit project as pregnancy outcomes were being audited and no intervention other than routine care according to existing clinical protocols was planned. Therefore, the project was exempted from formal ethics committee approval.

All pregnant women greater than 20-week gestation referred for public maternity care who resided within the postcodes 6001–6007, 6147, 6148, 6151, 6152, and 6155 within the North Metropolitan Health Service of the Western Australian Department of Health between July 1, 2010, to June 30, 2014, were included in the audit. Women with a history of preexisting diabetes mellitus (type 1 or 2) were specifically excluded from the project.

All women had an OGTT between 24 and 28 weeks of gestation in accordance with the existing clinical protocol [11].

The ADIPS 1999 criteria were used to diagnose GDM in the period from July 1, 2010, to December 31, 2011 [6]. Women had a 75-gram OGTT with glucose samples taken after an overnight fast and at 2 hr postprandially. GDM was diagnosed if the fasting glucose was ≥5.5 mmol/L (100 mg/dL) and/or the 2 h glucose was ≥8.0 mmol/L (~145 mg/dL) [6].

From January 1, 2012, to June 30, 2014, all patients were diagnosed with GDM using the IADPSG 2010 diagnostic criteria [4]. Women had a 75-gram OGTT with glucose samples taken after an overnight fast and at 1 hr and 2 hr postprandially. One or more abnormal values were needed for a diagnosis of GDM to be made: FBG > 5.0 mmol/L and/or 1-hour BSL > 10 mmol/L and/or 2-hour BSL ≥ 8.5 mmol/L.

The majority of OGTT in the audit period was performed at the Western Diagnostics Pathology laboratories. A small number (2.8%) was performed at other private accredited pathology providers.

All patients had their weight (kg) and height (m) recorded at their booking visit to calculate their body mass index (BMI). Patients with a BMI greater than 40 had their antenatal care at the local maternity hospital but were referred for delivery to the regional tertiary hospital. These patients remained within the audit study.

All pregnancies diagnosed with GDM across the audit period received identical clinical care according to a written protocol. This involved an initial consultation with a diabetic educator, dietician, and obstetric doctor. Patients commenced self-monitoring of blood sugar levels and adopted a diabetic diet. A review visit a fortnight later determined if medication was required in addition to diet.

Delivery outcomes were entered into a computerized database called Meditec by attending midwifery staff as part of routine practice. Delivery outcomes were subsequently extracted from Meditec, case note audit, and a postnatal clinical service for all women with GDM conducted by one author (Julie Quinlivan).

Predefined maternal outcomes were audited. These were mode of delivery, elective or emergency caesarean section, estimated blood loss, and 3rd or 4th degree perineal tear. Predefined newborn outcomes were audited. These were gestational age at birth, birthweight, birthweight >90th centile adjusted for gestational age, Apgar at 1 and 5 minutes, umbilical artery and vein pH, admission to Special Care Unit, and serious perinatal complications such as stillbirth, neonatal death, or birth trauma including shoulder dystocia.

A power calculation assumed that the change in incidence of GDM would be 30%, a conservative estimate based on the previous Australian and New Zealand studies [7, 8]. The baseline rate of GDM in the audit region was approximately 3.5%. Assuming a power of 80% and alpha error of 0.05, a sample of 10,994 women was required across the audit period to detect a change in incidence from 3.5 to 4.6%.

Data were presented as number and percentage for the incidence of GDM. Descriptive statistics of predefined clinical outcomes were compared using Student's *t*-test for continuous variables and Chi Square test or Fisher exact test for discrete data. A *p* value of 0.05 was considered significant.

## 3. Results

Of 10,296 women delivering in the audit period, antenatal OGTT results could be traced for 10,277 women (99.8%). The remaining 19 (0.2%) women did not have an antenatal OGTT, in violation of national clinical protocol. Of these women, 5 attempted an OGTT and were unable to complete the test due to nausea and/or vomiting. They subsequently declined a repeat test. The other 14 women either presented for care too late for testing or declined testing.

Table 1 summarizes the incidence of GDM under the two diagnostic criteria. The overall incidence was not significantly different with 3.4% diagnosed under the ADIPS 1999 criteria and 3.5% under the IADPSG 2010 criteria. In the subgroup of 342 women with a BMI > 40 (representing 3.3% of the study population) the incidence of GDM was 3.7% using ADIPS 1999 criteria and 8.5% using IADPSG 2010 criteria. This difference was not statistically significant (*p* = 0.11); however, the audit was not adequately powered to detect a difference in the subgroup of women with high BMI.

Across the audit period the proportion of women with GDM who required management with medication (metformin or insulin) in addition to diet was not significantly different (25% in women diagnosed by ADIPS 1999 criteria and 28% in women diagnosed by IADPSG 2010 criteria, resp.).

Delivery data for 10,277 (98%) women were available for audit through Meditec, case note audit, or the postnatal clinical service.

Table 2 summarizes predefined delivery outcomes. Babies born to women diagnosed with GDM according to the IADPSG 2010 criteria had significantly higher umbilical artery pH (7.28 versus 7.21; *p* = 0.01). They had a significant lower birthweight (3360 gms versus 3470 gms; *p* = 0.02) and birthweight above the >90th centile adjusted for gestational age (11% versus 18%; *p* = 0.04). Other predefined maternal and newborn outcomes were not significantly different between groups.

## 4. Discussion

The audit found no significant difference in the incidence of GDM before and after the introduction of the IADPSG 2010 criteria, with the overall incidence being low at 3.5%. Our results differ from the previous Australian and New Zealand studies [7, 8].

One explanation may be that prevalence of GDM in our region is low compared to many other sites. Our rate of 3.5% contrasts higher background rates in the sites involved in the HAPO trial where incidences ranged from 8 to 25% [12]. However, HAPO study sites were specifically included because of their high rates of GDM. They were tertiary sites where women with high BMI and other pregnancy complications were referred for antenatal and delivery management [5, 12]. Our study was based upon a geographical region rather than a hospital cohort and thus captured women of all risk levels, including a majority who were of “normal” risk, unlike the patient population within a tertiary centre.

A second explanation for the observed difference in outcome between our study and previous ones may be the racial mix of the population. Although our geographic maternity cohort reflected the wider Australian public maternity cohort in terms of maternal age and parity [13], racial background was overwhelmingly English speaking Caucasian.

A third explanation may be due to maternal obesity levels. Our geographic catchment has a low prevalence of overweight and obese patients compared to many sites. Lower obesity levels mean that the underlying risk of metabolic hyperglycaemia is lowered. Of note, in our subgroup of women with a BMI > 40 the incidence of GDM rose from 3.7% to 8.5% under the IADPSG 2010 criteria, more in line with studies elsewhere [7, 8].

As a secondary consideration, the adoption of the IADPSG 2010 criteria did not adversely impact upon our predefined maternal and newborn outcomes. There was a significant improvement in three newborn outcomes, being an increase in umbilical artery pH and a reduction in birthweight and birthweight >90th centile adjusted for gestational age. There were no significant changes in maternal outcomes. This provides reassuring safety data for the change.

The study had several strengths. Firstly, data were extracted from a defined geographic region before and after implementation of the IADPSG 2010 diagnostic criteria. Secondly, all women received treatment using identical clinical protocols throughout the audit period. Thirdly, there was high compliance with screening for GDM (99.8%) and ascertainment of outcome (98% of women). A study limitation is the low background incidence of GDM that limits generalizability to regions where incidence rates are higher. A second limitation is that only 3.3% of women presented with a BMI > 40. In this subgroup of women, the incidence of GDM was higher at 8.5%. Centres where the obstetric population has a higher incidence of obesity may report an increase in the incidence in GDM utilising the new diagnostic criteria. However, it is likely that this reflects a genuine increase in metabolic pathology, as obesity is a major risk factor for GDM and adverse pregnancy outcome [14].

## 5. Conclusion

The IADPSG used a consensus process to redefine GDM based on its association with adverse pregnancy outcomes. There has been controversy about the adoption of the new guidelines. However, in our audit study of 10,296 women, we observed no significant increase in the incidence of GDM. The adoption of the new criteria was associated with improvements in three newborn outcomes.

## Figures and Tables

**Table 1:** Incidence of GDM under ADIPS 1999 and IADPSG 2010 criteria.

	ADIPS	IADPSG	*p* value
All women	*N* = 3,553	*N* = 6,724	0.78
GDM	121 (3.4%)	236 (3.5%)	
No GDM	3,432 (96.6%)	6488 (96.5%)	

Women with BMI ≤ 40	*N* = 3446	*N* = 6489	0.86
GDM	117 (3.4%)	216 (3.3%)	
No GDM	3,329 (96.6%)	6,273 (96.7%)	

Women with BMI > 40	*N* = 107	*N* = 235	0.11
GDM	4 (3.7%)	20 (8.5%)	
No GDM	103 (96.3%)	215 (91.5%)	

**Table 2:** Delivery outcomes.

	GDM ADIPS *N* = 121	GDM IADPSG *N* = 236	*p* value
Maternal outcomes			
Maternal age (years) Mean (sd)	31.0 (5.3)	31.1 (5.6)	0.75
Parity Median (IQR)	1 (1–2.5)	1 (1–2.5)	0.34
Caesarean section *N* (%)	30 (25%)	64 (27%)	0.64
Blood loss (mL) Median (IQR)	300 (200–380)	300 (200–400)	0.25
Birth trauma (3rd/4th degree tear) *N* (%)	2 (2%)	5 (2%)	1.00

Newborn outcomes			
Gestational age (days) Mean (sd)	274 (7)	275 (6)	0.82
Birthweight (grams) Mean (sd)	3470 (345)	3360 (321)	0.02
Birthweight >90th centile for gestational age *N* (%)	22 (18%)	25 (12%)	0.04
Apgar 1 Mean (sd)	9 (8.25–9)	9 (9-9)	0.17
Apgar 5 Mean (sd)	9 (8.40–9)	9 (9-9)	0.21
Arterial cord blood Mean (sd)	7.21 (0.6)	7.28 (0.6)	0.01
Venous cord blood Mean (sd)	7.33 (0.2)	7.34 (0.2)	0.89
Admission to neonatal nursery *N* (%)	14 (12%)	28 (12%)	0.93
